# Alterations of the synapse of the inner retinal layers after chronic intraocular pressure elevation in glaucoma animal model

**DOI:** 10.1186/s13041-014-0053-2

**Published:** 2014-08-13

**Authors:** Hae-Young Lopilly Park, Jie Hyun Kim, Chan Kee Park

**Affiliations:** 1 Department of Ophthalmology and Visual Science, Seoul St. Mary’s Hospital, College of Medicine, The Catholic University of Korea, #505 Banpo-dong, Seocho-gu, Seoul 137-701, Korea

**Keywords:** Retinal ganglion cell, Glaucoma, Synapse

## Abstract

**Background:**

Dendrites of retinal ganglion cells (RGCs) synapse with axon terminals of bipolar cells in the inner plexiform layer (IPL). Changes in RGC dendrites and synapses between bipolar cells in the inner retinal layer may critically alter the function of RGCs in glaucoma. Recently, synaptic plasticity has been observed in the adult central nervous system, including the outer retinal layers. However, few studies have focused on changes in the synapses between RGCs and bipolar cells in glaucoma. In the present study, we used a rat model of ocular hypertension induced by episcleral vein cauterization to investigate changes in synaptic structure and protein expression in the inner retinal layer at various time points after moderate intraocular pressure (IOP) elevation.

**Results:**

Synaptophysin, a presynaptic vesicle protein, increased throughout the IPL, outer plexiform layer, and outer nuclear layer after IOP elevation. Increased synaptophysin after IOP elevation was expressed in bipolar cells in the innermost IPL. The RGC marker, SMI-32, co-localized with synaptophysin in RGC dendrites and were significantly increased at 1 week and 4 weeks after IOP elevation. Both synaptophysin and postsynaptic vesicle protein, PSD-95, were increased after IOP elevation by western blot analysis. Ribbon synapses in the IPL were quantified and structurally evaluated in retinal sections by transmission electron microscopy. After IOP elevation the total number of ribbon synapses decreased. There were increases in synapse diameter and synaptic vesicle number and decreases in active zone length and the number of docked vesicles after IOP elevation.

**Conclusions:**

Although the total number of synapses decreased as RGCs were lost after IOP elevation, there are attempts to increase synaptic vesicle proteins and immature synapse formation between RGCs and bipolar cells in the inner retinal layers after glaucoma induction.

## Background

Glaucoma represents a group of neurodegenerative diseases whose pathological hallmark is retinal ganglion cell (RGC) death [1]. In patients and experimental models of glaucoma, RGCs undergo apoptosis [2]-[4]. Many investigations have reported that chronic intraocular pressure (IOP) elevation, the clinical hallmark of glaucoma, induces decreases in soma size, dendrite shrinkage, and loss of RGC dendritic branches prior to apoptosis [5],[6]. Because dendritic structure determines the function of RGCs in visual processing and changes in dendritic morphology occur before cell death in the glaucomatous eye, studying dendritic changes in detail is important to understand the pathophysiology of glaucoma. RGC dendrites synapse on the axon terminals of bipolar cells in the inner plexiform layer (IPL) [7]. Early changes in RGC dendritic structure may have critical consequences for synaptic efficacy and may underlie functional deficits before RGC loss in glaucoma [8]. However, few studies have examined changes of the synapse between RGCs and bipolar cells in glaucoma [9].

Since axons of the adult central nervous system are unable to regenerate after injury, axonal outgrowth from RGCs slows substantially after birth [10]. However, unlike axons, RGC dendrites are a responsive element [11]. Dendrites have been shown to increase their dendritic receptive field and develop new dendritic branches after injury [12],[13]. Recent reports have demonstrated dendritic and synaptic plasticity of neurons in the adult central nervous system, including the retina [14]-[17]. For example, in retinal tissues obtained from patients with age-related macular degeneration, there is evidence of newly formed synapses between photoreceptors and bipolar cells in the outer retinal layers [18]. Although RGCs undergo apoptosis and dendrites degenerate after chronic IOP elevation in glaucoma, strengthening or increasing the number of synapses between RGCs and bipolar cells would delay functional loss. To further investigate the relevance of this concept to treatment, we must first understand how retinal synapse change in glaucoma. We used a glaucoma rat model in which ocular hypertension was induced by episcleral vein cauterization, which is similar to human glaucoma. Changes in synaptic elements in the inner retinal layers were examined at various time points after moderate IOP elevation.

## Results

### Presynaptic vesicles increased in the inner plexiform layer after IOP elevation

**Figure 1:**
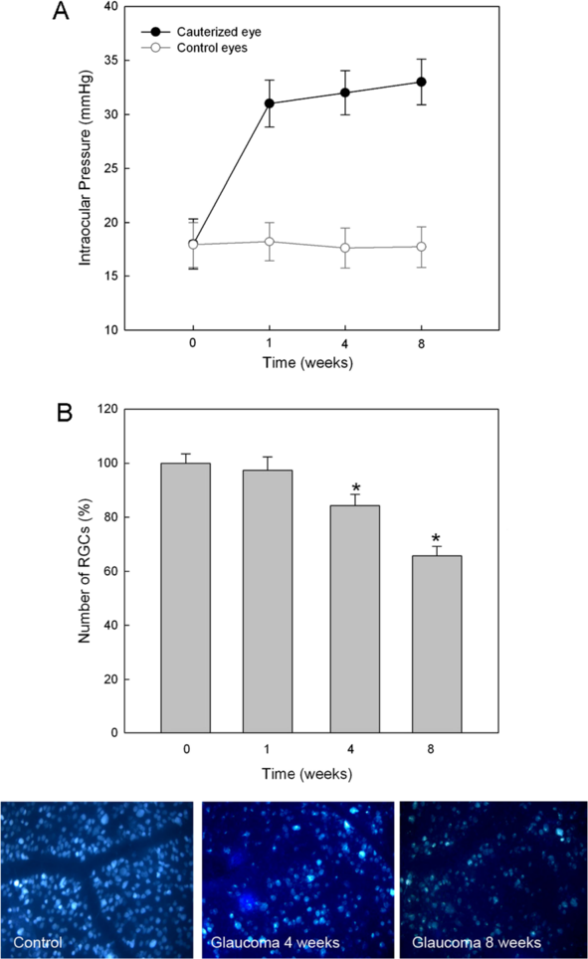
**Verifying intraocular pressure (IOP) elevation and reduction of retinal ganglion cells (RGCs) in a chronic hypertension glaucoma model.(A)** The change of IOP after cauterization in shown. The IOP of control and cauterized eyes was measured 0, 1, 4, and 8 weeks after cauterization. The IOP of the cauterized eyes was elevated to 30.4 ± 2.10 mmHg 1 week after cauterization and remained elevated during the 8 week experimental period (31.2 ± 2.26 mmHg). **(B)** Quantification of RGCs after elevation of IOP. The number of RGCs labeled with Fluorogold in control and cauterized eyes after 1, 4, and 8 weeks are shown. The loss of RGCs in the cauterized retinas relative to control (set at 100%) was approximately 97.0%, 84.6, and 67.1% at 1, 4, and 8 weeks after cauterization, respectively. Bar represents mean ± SD. Student’s t-test was used for statistical evaluation. *P < 0.05 compared to the control.

Cauterization of the episcleral vein induced sustained IOP elevation through the entire 8-week experiment was analyzed (Figure 1A). Specifically, IOP increased gradually after 1 week following surgery to 30.4 ± 2.10 mmHg from a basal value of 17.2 ± 2.26 mmHg. The average IOP in the cauterized eye over the 8-week period was 31.2 ± 2.26 mmHg. Control eyes that underwent sham surgery maintained normal IOP throughout the experiment.

RGCs were counted by retrograde labeling with Fluorogold stererotaxically injected into the superior colliculus at each time period. Approximately 112,000 RGCs were counted in normal control retinas at each time point. RGCs in retinas of the cauterized eyes were gradually lost following IOP elevation at 4 weeks and 8 weeks, the average number of retrograde-labeled RGCs decreased to 84.6% and 67.1%, respectively, of the average number of RGCs in normal control eyes (*P* <0.05, Figure 1B).

**Figure 2:**
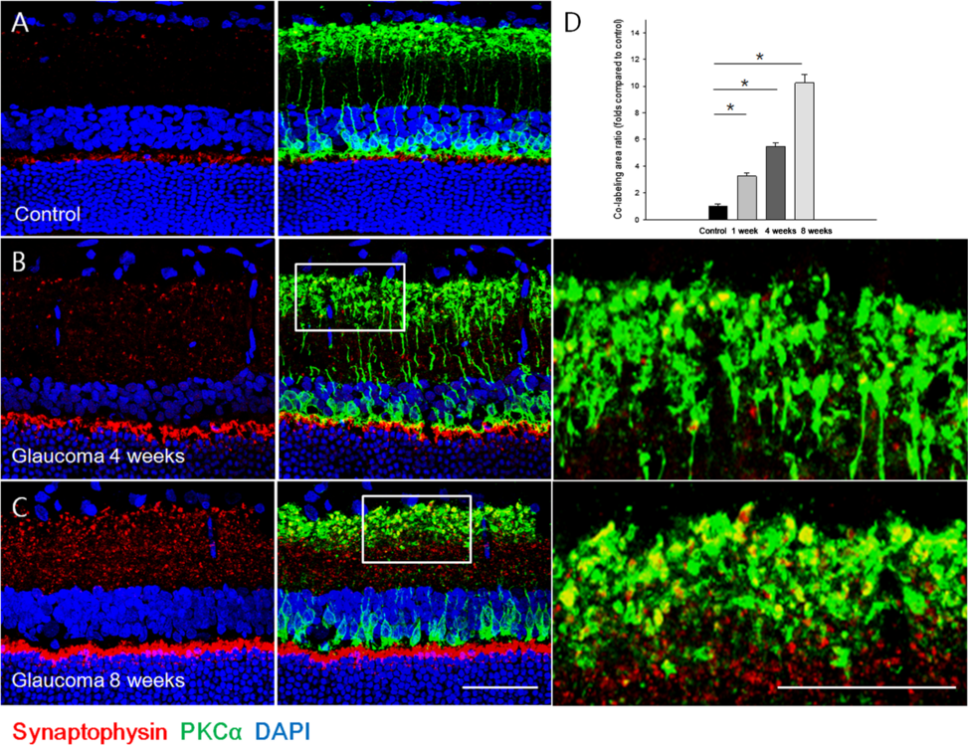
**Confocal micrographs of retinal sections double-stained for synaptophysin and PKCα, a bipolar cell marker.** In the control, synaptophysin was expressed in the innermost IPL and OPL **(A)**. After intraocular pressure elevation, expression of synaptophysin was increased throughout the IPL, OPL, and innermost ONL (**B** and **C**, left panel). Co-staining with PKCα revealed that upregulation of synaptophysin occurs in bipolar cells in the innermost IPL, where they synapse with retinal ganglion cells. Expression of synaptophysin and co-staining between synaptophysin and PKCα increased in the INL and OPL after 8 weeks of IOP elevation (**C**, middle panel) compared with 4 weeks (**B**, middle panel). Magnified confocal micrographs of the innermost inner plexiform layer in hypertensive eyes at 4 (**B**, right panel) and 8 weeks **(C**, right panel) post-surgery. Compared to the control (set to 1.0), the co-stained area significantly increased at 1, 4, and 8 weeks after IOP elevation **(D)**. *P < 0.05 compared with the control. Scale bars = 50 μm.

**Figure 3:**
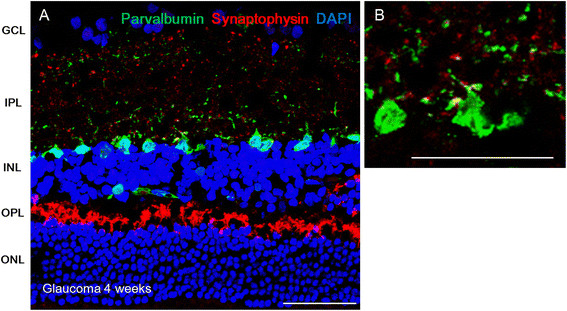
**Confocal micrographs of retinal sections double-stained for synaptophysin and parvalbumin, an amacrine cell marker.** At 4 weeks after intraocular pressure elevation, immunoreactivity for synaptophysin increased **(A)**. Little co-staining with parvalbumin was seen **(B)**. For cross-sectional immunohistochemical staining, n = 6 for control and n = 6 for glaucoma retinas at each time period; total n = 24. GCL, ganglion cell layer; IPL, inner plexiform layer; INL, inner nuclear layer; OPL, outer plexiform layer; ONL, outer nuclear layer. Scale bars = 50 μm.

After confirmation of IOP elevation and loss of RGCs in this chronic hypertensive glaucoma model, presynaptic vesicle proteins were assessed by immunolabeling for synaptophysin. Immunoreactivity for synaptophysin was increased throughout the IPL, OPL, and inner ONL after IOP elevation (Figure 2B and C) compared to normal controls (Figure 2A). Co-labeling with PKCα, a bipolar cell marker, revealed that bipolar cells in the innermost IPL were among the cells with upregulated synaptophysin. Immunoreactivity of synaptophysin increased in the IPL at each time point: 4 weeks (Figure 2B) and 8 weeks after IOP elevation (Figure 2C). Co-localization of synaptophysin and PKCα was also significantly increased in the innermost IPL at all time points (Figure 2D). Co-labeling with parvalbumin, an amacrine cell marker, showed that upregulation occurs to a lesser degree in amacrine cells (Figure 3). These data showed that IOP elevation increases presynaptic vesicles in the retina after IOP elevation for at least 8 weeks. These increases occur mainly in bipolar cells at the innermost IPL where bipolar cells synapse with RGCs.

### Increased presynaptic vesicles co-stained with RGC dendrites

**Figure 4:**
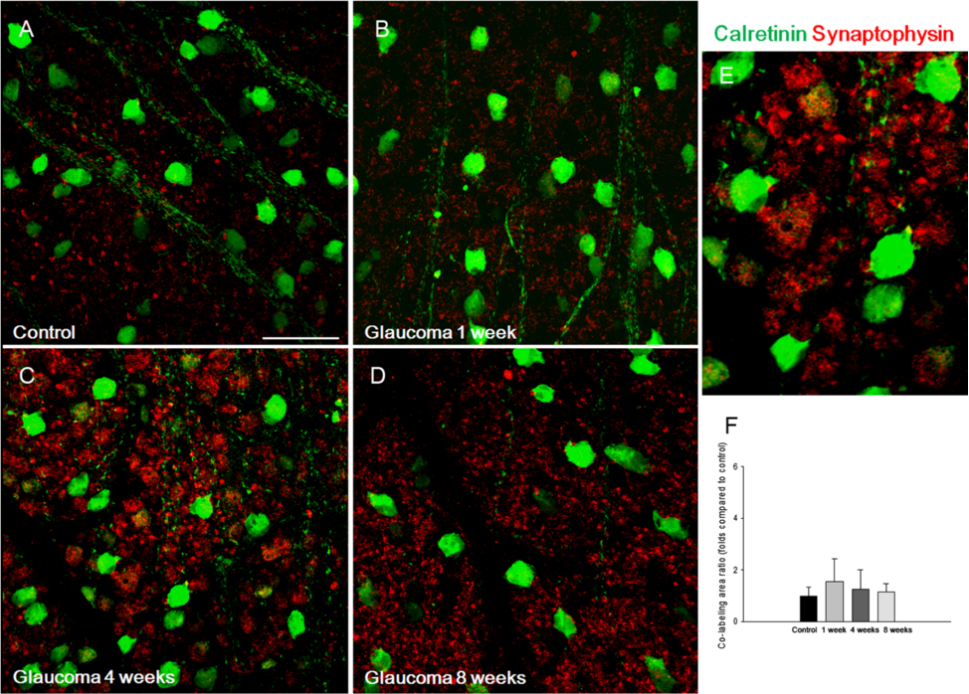
**Confocal micrographs of flat-mounted retinas double-stained for synaptophysin and calretinin, an amacrine cell and retinal ganglion cell marker, was focused on the border between the ganglion cell layer and the inner plexiform layer.** Celretinin stained the soma of the displaced amacrine cells and retinal ganglion cells in the ganglion cell layer. Immunoreactivity for synaptophysin increased at 1 **(B)**, 4 **(C)**, and 8 weeks **(D)** after intraocular pressure (IOP) elevation compared with the controls **(A)**. Magnification at 4 weeks after IOP elevation **(E)**. The area with both synaptophysin and calretinin co-staining did not change after IOP elevation (control set as 1.0, **F**). For flat mount immunohistochemical staining, n = 6 for control and n = 6 for glaucoma retinas at each time period; total n = 24. Scale bars = 50 μm.

**Figure 5:**
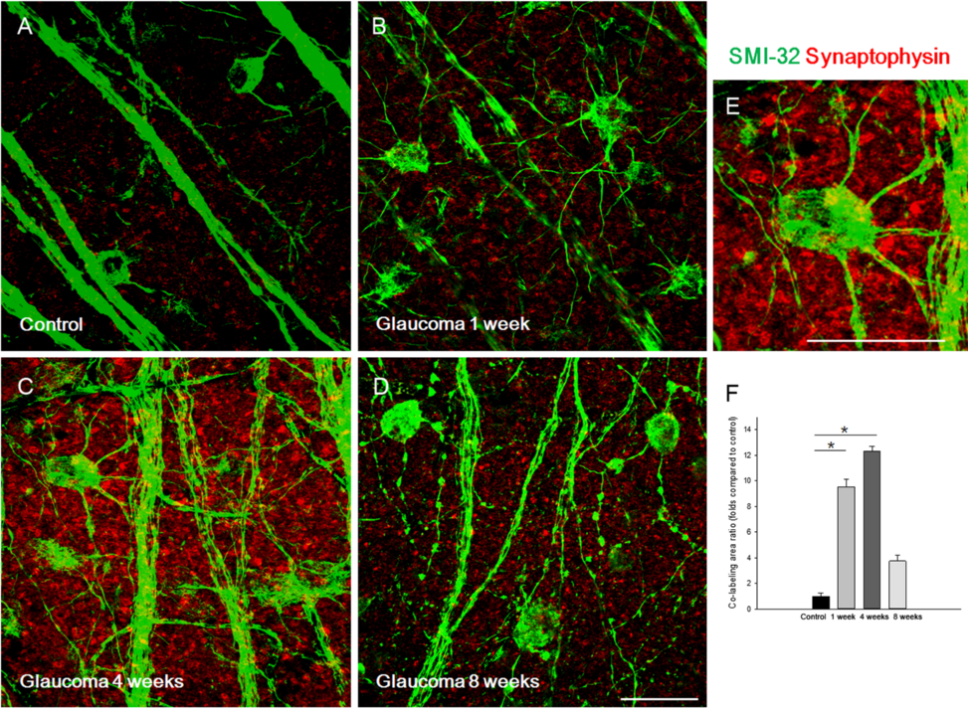
**Confocal micrographs of flat-mounted retinas double-stained for synaptophysin and SMI-32, a retinal ganglion cell (RGC) marker, focused on the border of the ganglion cell layer and the inner plexiform layer.** SMI-32 staining was seen in both the soma and dendrites of RGCs. Synaptophysin immunoreactivity increased at 1 **(B)**, 4 **(C)**, and 8 weeks **(D)** after intraocular pressure (IOP) elevation compared with controls **(A)**. SMI-32 immunoreactivity revealed increased and thickened RGC dendrites at 1 **(B)** and 4 weeks **(C)**, and rounded somas and decreased, beaded dendrites at 8 weeks after IOP elevation **(D)**. Magnification of flat mounts at 4 weeks after IOP elevation **(E)**. Co-staining between synaptophysin and SMI-32 showed that synaptophysin was expressed in the dendrites of RGCs. Co-stained areas significantly increased at 1 and 4 weeks after IOP elevation compared with the control (set to 1.0, **F**). For flat mount immunohistochemical staining, n = 6 for control and n = 6 for glaucoma retinas at each time period; total n = 24. Scale bars = 50 μm.

To determine whether increases in presynaptic vesicles originate from synapses between bipolar cells and RGCs, flat mount retinas were analyzed by immunostaining for markers of RGCs. Significant co-staining was observed between synaptophysin and calretinin, which stains the soma of amacrine cells and RGCs. Immunoreactivity for synaptophysin increased at 1 week (Figure 4B), 4 weeks (Figure 4C), and 8 weeks (Figure 4D) after IOP elevation compared to controls (Figure 4A). However, calretinin and synaptophysin signals were only found in small areas and did not significantly change after IOP elevation compared with controls (Figure 4F). SMI-32, a marker of neurofilaments, stains both the soma and dendrites of RGCs. Co-staining between SMI-32 and synaptophysin was significantly increased at 1 week (Figure 5B) and 4 weeks (Figure 5C) after IOP elevation compared with controls (Figure 5A). At 8 weeks, the RGC morphology changed in that the soma was rounded and the cells had few dendrites (Figure 5D). These results showed that increases in presynaptic vesicles are the result of changes within synapses between bipolar cells and RGCs, which are prominent at 1 week and 4 weeks after IOP elevation (Figure 5F).

### Presynaptic and postsynaptic vesicle proteins are all increased at a certain time period after IOP elevation

**Figure 6:**
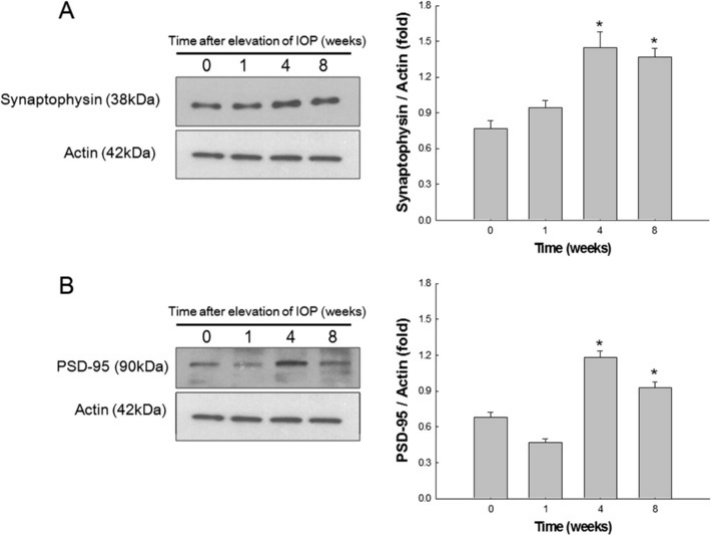
**The results of western blot analysis of synaptic vesicle proteins.** Synaptophysin, a presynaptic vesicle protein, was significantly increased in the retina at 4 and 8 weeks after intraocular pressure (IOP) elevation compared to controls **(A)**. PSD-95, a postsynaptic vesicle protein, was significantly increased in the retina at 4 and 8 weeks after IOP elevation compared to controls **(B)**. For western blot analysis, n = 6 for control and n = 6 for glaucoma retinas at each time period; total n = 48. Bar represents mean ± SD. Student’s t-test was used for statistical evaluation. *P < 0.05 compared to the control.

To quantify changes of synaptic vesicle proteins in the retina after IOP elevation, presynaptic and postsynaptic vesicle protein, synaptophysin and PSD-95, respectively, were examined using western blot analysis. Synaptophysin, a presynaptic vesicle protein, showed to increase until 4 weeks after IOP elevation and then slightly decrease at 8 weeks after IOP elevation, yet significantly increased when compared to controls (Figure 6A). PSD-95, a postsynaptic protein, showed to increase at 4 weeks and then slightly decrease at 8 weeks after IOP elevation, yet also significantly increased when compared to controls (Figure 6B). Quantification of synaptic vesicle proteins by western blot analysis shows that IOP elevation increases synaptic vesicle proteins in the retina. However, the increase in synaptic proteins seems to retard at 8 weeks after IOP elevation when RGC loss becomes prominent.

### IOP elevation decreases ribbon synapses, but structural alterations, between RGCs and bipolar cells

**Figure 7:**
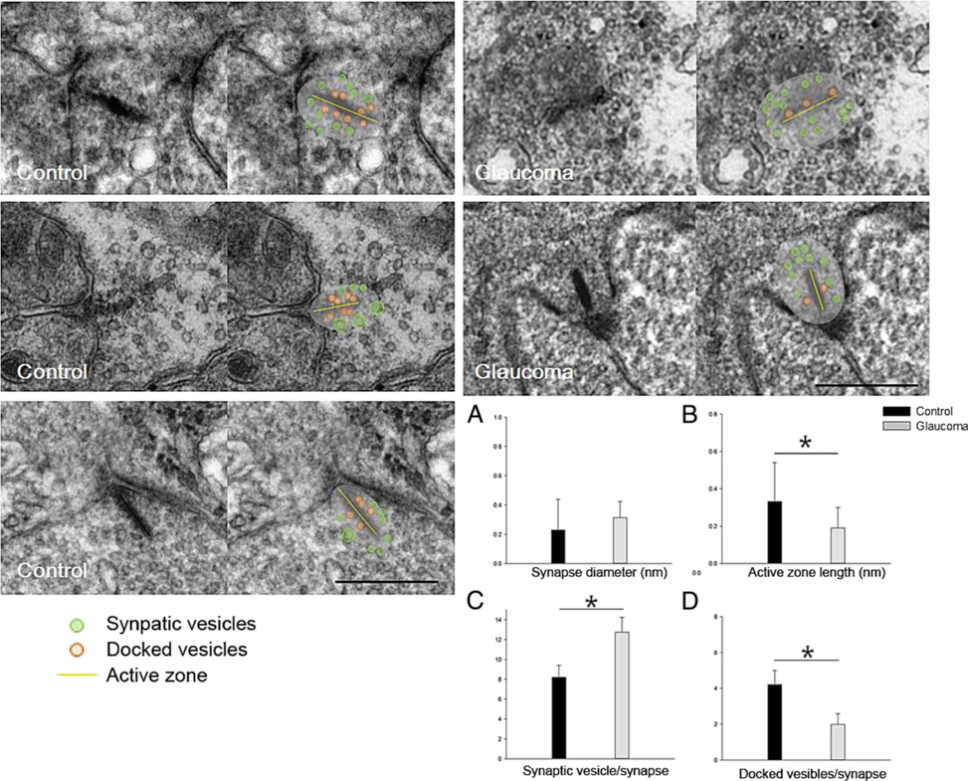
**Transmission electron micrographs of ribbon synapses between RGCs and bipolar cells in the inner plexiform layer.** After 8 weeks of intraocular pressure elevation, synapse diameter **(A)** and the number of synaptic vesicles **(C)** increased, and active zone length **(B)** and the number of docked vesicles **(D)** decreased. For electron microscopy, n = 6 for control and n = 6 for glaucoma retinas at 8 weeks, total n = 12. *P < 0.05 compared with the control. Scale bars = 0.5 μm.

The number of ribbon synapses in the IPL was quantified in retinal sections by transmission electron microscopy (EM). Before IOP elevation, ribbon synapses were occasionally seen (~7.4/50 μm^2^) in the IPL. However, after IOP elevation, the number of ribbon synapses decreased to ~3.2/50 μm^2^. We characterized synapse morphology by measuring presynapse profile diameter, active zone length, the number of morphologically apparent docked vesicles (<30 nm from the membrane), and the number of floating, undocked vesicles (Figure 7). Presynaptic profile diameter was larger in hypertensive retinas compared to controls, yet statistically not significant (Figure 7A). However, active zone length (control, 0.37 ± 0.16 nm; glaucoma, 0.19 ± 0.11 nm) and the number of docked synaptic vesicles per synapse significantly decreased (control, 4.02 ± 0.84; glaucoma, 2.05 ± 0.78), while the total number of synaptic vesicles per synapse significantly increased (control, 8.02 ± 1.24; glaucoma, 12.54 ± 1.98) with IOP elevation (Figure 7B-D). Thus, IOP elevation decreased the number of ribbon synapses and significantly changed their morphology.

## Discussions

We found through this work that synaptic vesicle proteins in RGCs and bipolar cells increase after chronic IOP elevation in a rat model of glaucoma. The expression of synaptophysin was increased in both bipolar cells and RGC dendrites. The number of the total ribbon synapses decreased in the IPL by EM; however, their morphology suggests immature, newly formed synapses between RGCs and bipolar cells. These observations may indicate that loss of RGC due to chronic IOP elevation leads to increases in attempt to form new synaptic contacts between RGCs and bipolar cells.

Synapse between bipolar cells and RGCs may be important in glaucoma since the main cell death occurs in the RGCs. Presynaptic protein in the bipolar cells, analyzed using synaptophysin, gradually increased in the IPL throughout the 8-week experiment period. However, postsynaptic protein in RGCs, analyzed using PSD-95, increased at 4 weeks and then decreased at 8 weeks. This may be due to significant RGC loss at 8 weeks after IOP elevation.

Studies that focused on potential alterations in synapses generally have compared the numbers of synapses, as well as their structure [19], including the number of synaptic vesicles, the length of the active zone, and the proportion of vesicles that are docked or floating [19]. As synapses between neurons mature during development, they become smaller and the active zone becomes longer and thicker [20],[21]. The number of total synaptic vesicles may or may not change; however, the number of docked vesicles increases [22],[23]. These changes occur in both the pre- and postsynaptic elements [23]. By EM, we found that following glaucoma induction, ribbon synapses between RGCs and bipolar cells had increased diameter, shorter active zone lengths, and a greater number of synaptic vesicles, but fewer docked vesicles per synapse than those in control retinas. These features indicated immature, newly formed ribbon synapses in the inner retinal layers, which may suggest a compensatory mechanism to restore synaptic connections between RGCs and bipolar cells lost due to RGC apoptosis.

Previously, dendritic shrinkage and synaptic disconnection of RGCs in the early phase of ocular hypertension have been reported [9],[24]. However, a study comparing dendritic changes in RGCs following ocular hypertension and optic nerve crush reported that unlike optic nerve crush, RGCs in hypertensive eyes maintained their dendritic structure through the 6 week observation period [24], and a selected few RGCs had greater dendritic complexity than that of control eyes [24]. Together with our findings, there results suggest that glaucomatous pathology induces morphologic plasticity of RGCs [25].

Our previous studies showed that RGCs express phosphorylated Akt in the early phase of IOP elevation and this response is enhanced after upregulation of brain derived neurotrophic factor (BDNF) [26],[27]. The BDNF and its downstream receptor signaling including Akt are consistently reported to contribute to synapse formation during development [28]. Recently, loss of photoreceptors showed to lead upregulation of synaptic proteins in the retina representing synaptic plasticity in response to injury [29]. Together, there may also be possibilities to change the synaptic connections between neuronal cells in the adult retina after injury.

Hypertension-induced morphological changes in RGCs in 4 weeks post-induction, as revealed by SMI-32 immunostaining, include more complex dendritic structures and more numerous axon bundles in the GCL than in controls or during early stages of hypertension. During this stage, co-staining of SMI-32 and synaptophysin, which indirectly reveals synaptic contact areas, was also increased. These observations also suggest that RGC loss is compensated by the expansion of the dendritic range to occupy abandoned receptive fields.

## Conclusions

Our study demonstrates that expression of synaptic vesicle proteins increases in the inner retinal layers after chronic IOP elevation. Both presynaptic and postsynaptic vesicle proteins increased between RGCs and bipolar cells. The total number of synapses decreased as RGCs were lost after IOP elevation; however, the EM findings suggested immature synapse that might indicate newly formed synapses in the inner retinal layers.

## Methods

### Animals

Adult male Sprague–Dawley rats (7–8 weeks old, 250–300 g) were used in this study. Six animals were included in each group for each experimental procedure. For transmission EM, three animals were used for each experimental period; the total number of animals used was 96. All animal experiments complied with the ARVO statement for the Use of Animals in Ophthalmic and Vision Research, the regulations of the Catholic Ethics Committee of the Catholic University of Korea, Seoul, and the National Institutes of Health Guide for the Care and Use of Laboratory Animals (NIH Publications, no. 80–23, revised 1996). All efforts were made to minimize suffering and the number of animals used. Prior to surgery, rats were anesthetized by intraperitoneal injection of 50 mg/kg tiletamine plus zolazepam (Zoletil; Virbac) and 15 mg/kg xylazine hydrochloride (Rompun; Bayer). Three episcleral veins were cauterized by a standard technique previously described [30]. Briefly, before the procedure, IOP was measured with a tonometer (Tono-Pen; Mentor O&O Inc.). A small conjunctival incision was made in each quadrant at the limbus, and the extraocular muscles were isolated. Four major limbal draining veins were identified based on their deep location under the rectus muscles, relative immobility, large caliber, and branching pattern. Among them, three episcleral veins, specifically two dorsal episcleral veins under the superior rectus muscle and one temporal episcleral vein under the lateral rectus muscle, were cauterized using a surgical microscope (Olympus) and a cautery with a 30-gauge tip (Bovie Co.) to avoid possible damage to the neighboring sclera. Planar ophthalmoscopy confirmed normal perfusion of the retina. After surgery, chloramphenicol eye drops and oxytetracycline ointment were applied to the eyes. Only eyes that did not suffer scleral burns with subsequent necrosis or any surgical complications were used. IOP was measured directly and carefully by a tonometer (Tono-Pen) after topical anesthetization with proparacaine hydrochloride ophthalmic solution (Alcane; Alcon Laboratories). Animals were kept as calm as possible to minimize effects on the IOP readings. Experimental analyses were performed 1, 4, and 8 weeks after cauterization. Eyes without sustained IOP throughout the 8-week experiment period were excluded.

### Tissue preparation

At each time point, for immunohistochemical analysis, eyes were quickly enucleated and dissected, and the posterior eye cups were placed in chilled fixative (4% paraformaldehyde in 0.1 M phosphate buffer [PB], pH 7.4). Isolated retinas were fixed in the same fixative for 2 h at 4°C. After washing several times, fixed retinas were cryoprotected in 30% sucrose containing 0.1 M PB for 6 h at 4°C and stored in this buffer at - 70°C. For EM, retinal tissues were fixed in glutaraldehyde. For western blot analysis, retinal tissues were quickly dissected, frozen in liquid nitrogen, and stored at - 70°C.

### Counting of RGCs

For retrograde labelling of RGCs, 4 days before sacrifice, Fluorogold (FG; Fluorochrome; 2 μL of 5% solution) was introduced stereotaxically either unilaterally or bilaterally into the superior colliculus. Briefly, after fixing the head of anesthetized rats in a stereotaxic apparatus (Stoelting) with the skull held horizontally, FG was injected into the superior colliculus using the following coordinates: 6 mm posterior to the bregma, 1.2 mm lateral to the midline, and 3.8–4.2 mm deep from the top of the skull. These tracers are taken up by the axon terminals of RGCs in the superior colliculus and transported to the somas in the retina. Immediately after sacrifice, the superior side of each eye was marked for orientation, and both eyes were enucleated. The anterior segments were removed, and the posterior segments were fixed in 4% paraformaldehyde in 0.1 M PB, pH 7.4, for 30 min. The retina was then isolated, divided into four equal quadrants, and flat-mounted on slides. RGCs were counted as previously reported [30]. Briefly, each retinal quadrant was divided into central, middle, and peripheral regions (1, 2, and 3 mm from the optic disc, respectively) and microscopic fields measuring 200 × 250 μm^2^ were selected. Labeled ganglion cells were counted at 200x magnification in four central regions, eight middle regions, and twelve peripheral regions in the four quadrants of the retina. Corresponding regions from each retina of experimental and control groups were used for counting.

### Transmission electron microscopy

Electron microscopy was conducted using retinal sections from vein-cauterized and sham-operated controls at 8 weeks post-surgery (three rats per group). Ten fields of each eye were examined. Retinal sections (100 μm) were cut with a vibratome, postfixed with 4% glutaraldehyde in 0.1 mmol/L cacodylate buffer (pH 7.4) for 1 h, and then with 1% osmium tetroxide in 0.1 mmol/L cacodylate buffer for 2 h. After rinsing with distilled water, sections were treated with 1% aqueous uranyl acetate overnight, dehydrated in ethanol solutions of increasing concentration, up to 100%, followed by dry acetone, and then embedded in Durcupan ACM. Ultrathin sections (0.1 μm) were cut and mounted on Formvar-coated slot grids, stained with 3% lead citrate, and examined with a Zeiss transmission EM (Zeiss). Ribbon synapses were counted in 50 micrographs (2,500 μm^2^ total). Synaptic parameters and dimensions of scanned areas were quantified directly from the microscope’s calibrated scale.

### Immunohistochemistry

To evaluate synaptic vesicles, retinal expression of synaptophysin was assessed. For fluorescence staining, samples were pre-embedded in 3% agar in deionized water. Vibratome sections (50 μm) were collected and washed several times in PBS. Sections were incubated in 10% normal donkey serum in PBS for 1 h at room temperature to block nonspecific binding activity, then with anti-rabbit synaptophysin (Cell signaling, Danvers, MA, USA) overnight at 4°C. After several washes with PBS, sections were incubated with goat anti-rabbit Alexa 546 (Molecular Probes). For double-labeling studies, sections were incubated with anti-mouse PKCα (Santa Cruz Biotechnology, Santa Cruz, CA), parvalbumin (Sigma, St. Louis, MO), calretinin (Millipore, Temecula, CA, USA), or SMI-32 (Covance, Emeryville, CA, USA) in 0.1 M PBS containing 0.5% Triton X-100 overnight at 4°C, rinsed for 30 min with 0.1 M PBS, and incubated with goat anti-mouse Alexa 488 (Molecular Probes) for 1 h 30 min at room temperature. After further washes in 0.1 M PB for 30 min, the sections were mounted using VectaShield Mounting Medium with DAPI (Vector Laboratories, H-1200). Slides were washed, covered with coverslips, and examined by confocal laser scanning microscopy (Zeiss LSM 510; Carl Zeiss Co. Ltd.).

### Western blot analysis

Control and injured retinas were homogenized in RIPA buffer (1% Triton X-100, 5% SDS, 5% deoxycholic acid, 0.5 M Tris–HCl pH 7.5, 10% glycerol, 1 mM EDTA, 1 mM phenylmethylsulfonyl fluoride [PMSF], 5 μg/ml aprotinin, 1 μg/ml leupeptin, 1 μg/ml pepstatin, 200 mM sodium orthovanadate, and 200 mM sodium fluoride). Tissue extracts were incubated for 10 min on ice and clarified by centrifugation at 10,000 x g for 25 min at 4°C. Total protein in retinal extracts was measured using a standard BCA assay (Pierce). Retinal extracts (40 μg total protein) were resuspended in 5 x sample buffer (60 mM Tris–HCl pH 7.4, 25% glycerol, 2% SDS, 14.4 mM 2-mercaptoethanol, 0.1% bromophenol blue) at a 4:1 ratio, boiled for 5 min, and resolved by SDS-PAGE. Proteins were transferred onto a nitrocellulose membrane, and blots were stained with Ponseau S (Sigma, St. Louis, MO, USA) to visualize the protein bands and ensure equal protein loading and uniform transfer. Blots were washed and blocked for 45 min with 5% non-dried skim milk in TBST buffer (20 mM Tris–HCl pH 7.6, 137 mM NaCl, and 0.1% Tween 20). Blots were then probed for 24 h using antibodies against synaptophysin (Cell signaling, Danvers, MA, USA), PSD-95 (Thermo, Rockford, IL, USA), and Actin (Sigma, St. Louis, MO, USA). Blots were then probed with horseradish-peroxidase (HRP)-conjugated goat anti-rabbit secondary antibody. Bound antibodies were detected using an enhanced chemiluminescence system (Amersham) and X-ray film. Relative intensity was measured using an ImageMaster®VDS (Pharmacia Biotech) and the fold changes in these protein levels are indicated below the blot. Results are representative of five independent experiments. Data are expressed as mean ± SD.

### Statistical analysis

All data are expressed as means ± SD. Comparisons between time points or with the controls were performed using the Student’s t-test and multiple comparisons using the Scheffe’s post hoc method. Differences with *p* < 0.05 were considered statistically significant.

## Abbreviations

RGC: Retinal ganglion cells

IOP: Intraocular pressure

GCL: Ganglion cell layer

IPL: Inner plexiform layer

INL: Inner nuclear layer

OPL: Outer plexiform layer

PKCα: Protein kinase C alpha

## Competing interests

The authors declare that they have no competing interest.

## Authors’ contributions

Design and conduct of the study (CKP, JHK, HYP), collection (CKP, JHK, HYP), management (CKP, JHK, HYP), Analysis (CKP, JHK, HYP), interpretation of the data (CKP, JHK, HYP), preparation (JHK, HYP), review or approval of the manuscript (CKP, JHK, HYP). All authors read and approved the final manuscript.
